# Strain-dependent mutational effects for *Pepino mosaic virus* in a natural host

**DOI:** 10.1186/s12862-017-0920-4

**Published:** 2017-03-06

**Authors:** Julia Minicka, Santiago F. Elena, Natasza Borodynko-Filas, Błażej Rubiś, Beata Hasiów-Jaroszewska

**Affiliations:** 1 Department of Virology and Bacteriology, Institute of Plant Protection–National Research Institute, Poznan, Poland; 2 Instituto de Biología Molecular y Celular de Plantas, Consejo Superior de Investigaciones Científicas–Universidad Politécnica de Valencia, València, Spain; 3 Instituto de Biología Integrativa y de Sistemas, Consejo Superior de Investigaciones Científicas-Universitat de València, València, Spain; 4 The Santa Fe Institute, Santa Fe, New Mexico USA; 5 Department of Clinical Chemistry and Molecular Diagnostics, Poznan University of Medical Sciences, Poznan, Poland

**Keywords:** Epistasis, Mutational fitness effect, *Pepino mosaic virus*, Real-time quantitative PCR, Site-directed mutagenesis, Virus accumulation, Virus evolution

## Abstract

**Background:**

*Pepino mosaic virus* (PepMV) is an emerging plant pathogen that infects tomatoes worldwide. Understanding the factors that influence its evolutionary success is essential for developing new control strategies that may be more robust against the evolution of new viral strains. One of these evolutionary factors is the distribution of mutational fitness effect (DMFE), that is, the fraction of mutations that are lethal, deleterious, neutral, and beneficial on a given viral strain and host species. The goal of this study was to characterize the DMFE of introduced nonsynonymous mutations on a mild isolate of PepMV from the Chilean 2 strain (PepMV-P22). Additionally, we also explored whether the fitness effect of a given mutation depends on the gene where it appears or on epistatic interactions with the genetic background. To address this latter possibility, a subset of mutations were also introduced in a mild isolate of the European strain (PepMV-P11) and the fitness of the resulting clones measured.

**Results:**

A collection of 25 PepMV clones each containing a single nucleotide nonsynonymous substitution was created by site-directed mutagenesis and the fitness of each mutant was determined. PepMV-P22 genome showed a high degree of robustness against point mutations, with 80% of mutations being either neutral or even beneficial and only 20% being deleterious or lethal. We found that the effect of mutations strongly depended on the gene in which they were introduced. Mutations with the largest average beneficial effects were those affecting the *RdRp* gene, in contrast to mutations affecting *TGB1* and *CP* genes, for which the average effects were deleterious. Moreover, significant epistatic interactions were observed between nonsynonymous mutations and the genetic background, meaning that the effect of a given nucleotide substitution on a particular genomic context cannot be predicted by knowing its effect in a different one.

**Conclusions:**

Our results indicated that PepMV genome has a surprisingly high robustness against mutations. We also found that fitness consequences of a given mutation differ between the two strains analyzed. This discovery suggests that the strength of selection, and thus the rates of evolution, vary among PepMV strains.

## Background

The main source of genetic variation in RNA viruses is spontaneous mutation, which is introduced into the genome as a result of a lack of proofreading activity of viral RNA-dependent RNA polymerases [1]. As a consequence, RNA viruses have a higher rate of spontaneous mutations than DNA viruses, ranging from 0.01 to 2 mutations per genome and generation [2]. The supplying of numerous mutations provides an opportunity for better adaptation to environmental perturbations [3]. However, mutation is a shortsighted process, so the organisms cannot enjoy the long-term benefits without incurring into short-term negative effects. Such a negative consequence is the reduction in fitness due to the accumulation of deleterious mutations in absence of purifying selection [4–6]. Therefore, the evolutionary success of viruses and their survival depend on avoiding the negative consequences of arising deleterious mutations [7].

The effect of mutations can be lethal, neutral, deleterious or beneficial. Neutral mutations accumulate most often in the genome, as a result of a poor operation or non-action of selection upon them. Nevertheless, the fitness effect of all kinds of mutations are important to adaptation. Neutral and deleterious mutations provide a genetic background, which is important for the fixation of future beneficial mutations. The final effect of beneficial mutations depends on the presence of epistatic interactions with others (neutral and deleterious) mutations. However, during population bottlenecks, it is also possible to fix deleterious mutations, that may be subsequently removed by purifying selection or, alternatively, whose effects should be compensated by other mutations arising during subsequent expansion of the population or by reversion to the wild type allele [2]. Therefore, the distribution of mutational fitness effects (DMFE), which is the fraction of all possible mutations that are beneficial, neutral, deleterious and lethal, is pivotal for understanding the evolutionary biology of the viruses.

In the last fifteen years, considerable effort has been devoted into describing the properties of the DMFE for some animal [8–11] and plant [12, 13] RNA viruses, RNA and DNA bacteriophages [14, 15], as well as for several eukaryotes such as *Drosophila melanogaster* [16], *Caenorhabditis elegans* [17] and *Saccharomyces cerevisiae* [18], just to mention a few. For the case of viruses, some general properties can be drawn from these studies [19], which all share similar experimental approaches: DMFEs are strongly skewed towards deleterious effects, with a large fraction of mutations being lethal in the assayed conditions. For example, it has been shown for *Vesicular stomatitis virus* (VSV) [9] and *Tobacco etch virus* (TEV) [12] that lethal and deleterious effects add up to 70–80% of all single mutations analyzed, with average deleterious effects being in the range of 11–13%. It is generally accepted that the beneficial effect of mutation is rare and happens no more than once out of a thousand cases [20–22], though their average fitness benefits may be as large as 30% (e.g., for VSV [22]). Taken together, the abundance of deleterious and lethal mutations and the scarcity of beneficial mutations suggest that in the short-term virus evolution should be dominated by the combined action of mutation, drift and purifying selection [23], while the role of beneficial mutations may be relevant only at the host-population level and long-term periods, where the chances of such mutations to arise and spread until fixation may be larger.

Multiple mutations may act independently to determine the fitness of these strains or interact in an epistatic manner [24]. In the context of adaptive landscapes, epistasis determines the ruggedness and neutrality of the landscape, with themselves determine evolvability, the reproducibility of evolution, and the accessibility of high fitness optima from low fitness spots in the landscape [25]. Only very recently the topological properties of fitness landscapes have been empirically explored for an RNA virus, TEV, showing that, at least for this plant virus, fitness landscapes are highly rugged and that ruggedness depends on the particular host wherein fitness was evaluated [26–30].

In this study, we analyzed the DMFE of *Pepino mosaic virus* (PepMV) on tomato, which is the only natural host for this virus so far. PepMV belongs to the genus *Potexvirus* in Alfaflexiviridae family and is a highly infective and virulent pathogen included in the EPPO A2 list of the European and Mediterranean Plant Protection Organization (EPPO). PepMV mainly infects tomato plants, as well as other hosts from the *Solanaceae* family [31]. In recent years, the virus has spread quickly in the United States, Chile and Europe, causing significant losses in quality and yield potential [32–39]. The PepMV genome consists of a single, positive-sense RNA molecule of approximately 6.4 kb encoding five genes: RNA dependent RNA polymerase (*RdRp*), movement proteins (*TGB1*, *TGB2* and *TGB3*) and coat protein (*CP*). Currently, the virus is classified into five strains (consider as a group of genetically similar isolates): European (EU), Peruvian (LP), southern Peruvian (PES), American 1 (US1) and Chilean 2 (CH2) [40, 41]. Currently, isolates belonging to the CH2 strain predominate in European and the United States populations of the virus [40–42]. Although the nucleotide sequence identity between isolates closely related to each other ranges 99%, they cause diverse disease symptoms. It has been previously shown that single nucleotide substitutions A199G (TGB3/K67E) in *TGB3* and G463A (CP/E155K) and A497G (CP/D166G) in *CP* genes play a role in the development of necrotic and yellowing symptoms, respectively [43–45]. Furthermore, a high number of genetic variants were identified during long-term experimental evolution (20 serial passages in five evolutionary lineages) of PepMV in different hosts [46]. After 18 passages in one of the hosts (tomato cultivar Beta Lux) necrotic symptoms appeared in two independent evolutionary lineages leading to death of the plants. This suggests that the virus can evolve rapidly and changes in the genome may have dramatic effect on its biological properties [46]. Moreover, PepMV populations conform to the viral quasispecies model, by being constitute a cloud of phylogenetically related, though genetically different variants, under the mutation-selection balance [47]. This type of population structure replicates near the maximum error rate compatible with the maintenance of the encoded genetic information [48]. The quasispecies nature of RNA viruses is linked with a high adaptive potential and selection of variants with the highest fitness in new environments. It may have great impact on viral virulence and pathogenicity including the appearance of resistance-breaking strains or the acquisition of new hosts [49, 50].

The aim of this study is threefold. Firstly, we characterized the DMFE associated to nonsynonymous mutations on the PepMV-P22 mild isolate of the CH2 strain. Fitness effects were measured on tomato. Secondly, to analyze whether fitness effects of individual mutations were strain-dependent, *i.e*. whether epistasis exists between point mutations and the genetic background, we measured the fitness effects of a subset of the mutations in the PepMV-P11 mild isolate of the EU strain. Thirdly, we sought to describe the topography of the local fitness landscape defined by the previously described pair of mutations in the *CP* gene that are responsible for the appearance of new symptoms.

## Methods

### Viral material

All experiments were performed on mild isolates of PepMV from CH2 and EU strains, designated as PepMV-P22 and PepMV-P11, respectively. Complete genome sequences of both isolates had previously been submitted to the GenBank, under accession numbers HQ650560 and JN133846, respectively. Plasmids bearing a full-length cDNA clone of the RNA genome of PepMV-P22 and PepMV-P11 were constructed according to previously published procedure [43, 51].

### Site-directed mutagenesis

**Fig. 1:**
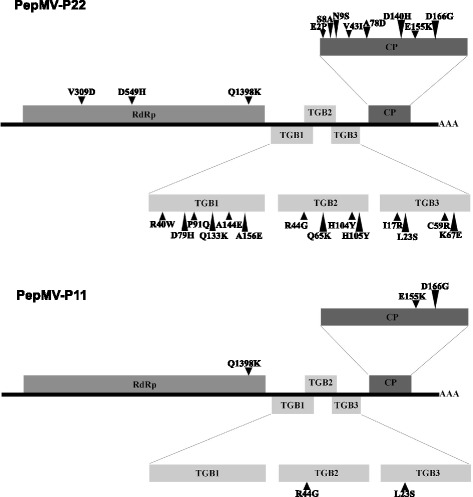
Genome organization of PepMV and location of the single nucleotide substitutions introduced by site-directed mutagenesis

Twenty-five single nucleotide nonsynonymous substitutions were introduced into the genome of PepMV-P22 by site-directed mutagenesis using a Phusion Site-Directed Mutagenesis Kit (ThermoFisher Scientific, Wilmington, DE, USA) according to the manufacturer’s protocol. Twenty-two were randomly chosen and three were previously described [43, 45]. Five of these mutations (three random and two previously described as a determinant of yellowing symptoms) were also introduced into the genome of PepMV-P11 (Table 1 and Fig. 1). In addition, mutations resulting in the amino acid substitutions CP/E155K and CP/D166G were introduced simultaneously into the mild EU isolate PepMV-P11 to characterize their epistatic interactions (*i.e*. the topography of the local fitness landscape). All primers were designed to introduce a single nucleotide substitution within the individual codons (Table 1). Furthermore, mutations were introduced across all regions of the genome. Each PCR reaction was performed in a volume of 50 μl and contained 0.1 ng of plasmid DNA, 10 μl of Phusion HF Buffer (5×), 1 μl of dNTPs (200 μM each), 1 μl of each primer (10 μM) and 0.5 μl of Phusion Hot Start DNA Polymerase (2 U/μl). Amplifications were carried out in a Thermal Cycler (Biometra, Goettingen, Germany) and consisted of initial denaturation at 98 °C for 30 s, followed by 30 cycles of 15 s at 98 °C, 40 s at 60 °C and 6 min at 72 °C. The resulting PCR products were then linearized with *Dpn*I (ThermoFisher Scientific, Wilmington DE, USA) for two hours at 37 °C and purified using NucleoSpin® Gel and PCR Clean-up (Macherey-Nagel, Düren, Germany) according to the manufacturer’s protocol. All products were ligated using 5 μl of Quick Ligation Reaction Buffer (2×) and 0.5 μl of Quick T4 DNA Ligase for 5 min at room temperature (25 °C) and transformed to One Shot Top 10 chemically competent *Escherichia coli* (ThermoFisher Scientific, Wilmington DE, USA). Plasmid DNA were isolated from individual colonies using an Insorb Spin Plasmid Mini Two kit (Stratec Molecular, Berlin, Germany). The presence of a specific mutation was confirmed by sequencing using a Beckman Coulter CEQ8000 sequencer (Beckman Coulter, London, UK).

**Table 1:** PepMV strains used in the study and changes in the genomes

Strain	Isolate	Gene	Location in particular gene^a^	Codon substitution	Amino acid change	Effect^b^	*t*-test *P*
CH2	P22	*RdRp*	926 (309)	GTC-GAC	V-D	Beneficial	<0.0001
CH2	P22	*RdRp*	1647 (549)	GAC-CAC	D-H	Neutral	0.0686
CH2	P22	*RdRp*	4192 (1398)	CAA-AAA	Q-K	Neutral	0.1904
CH2	P22	*TGB1*	118 (40)	AGG-TGG	R-W	Neutral	0.3804
CH2	P22	*TGB1*	235 (79)	GAC – CAC	D-H	Neutral	0.0920
CH2	P22	*TGB1*	272 (91)	CCU-CAU	P-H	Neutral	0.8217
CH2	P22	*TGB1*	431 (144)	GCA-GAA	A-E	Lethal	<0.0001
CH2	P22	*TGB1*	467 (156)	GCA-GAA	A-E	Lethal	<0.0001
CH2	P22	*TGB2*	130 (44)	AGA – GGA	R-G	Neutral	0.0203
CH2	P22	*TGB2*	193 (65)	CAA-AAA	Q-K	Beneficial	<0.0001
CH2	P22	*TGB2*	310 (104)	CAT-TAT	H-Y	Beneficial	0.0106
CH2	P22	*TGB2*	313 (105)	CAT-TAT	H-Y	Beneficial	0.0004
CH2	P22	*TGB3*	50 (17)	ATA-AGA	I-R	Neutral	0.0858
CH2	P22	*TGB3*	68 (23)	TTA-TCA	L-S	Neutral	0.2530
CH2	P22	*TGB3*	175 (59)	TGT - CGT	C-R	Neutral	0.0237
CH2	P22	*TGB3*	199 (67)	AAA- GAA	K-E	Neutral	0.9274
CH2	P22	*TGB3*	397 (133)	CAG-AAG	Q-K	Beneficial	0.0006
CH2	P22	*CP*	5 (2)	GAA-GCA	E-A	Deleterious	0.0026
CH2	P22	*CP*	22 (8)	TCT-GCT	S-A	Neutral	0.0113
CH2	P22	*CP*	26 (9)	AAC-AGC	N-S	Lethal	<0.0001
CH2	P22	*CP*	127 (43)	GTC-ATC	V-I	Neutral	0.0420
CH2	P22	*CP*	233 (78)	GCC-GAC	A-D	Lethal	<0.0001
CH2	P22	*CP*	418 (140)	GAC-CAC	D-H	Neutral	0.0827
CH2	P22	*CP*	463 (155)	GAA-AAA	E-K	Neutral	0.0183
CH2	P22	*CP*	497 (166)	GAT-GGT	D-G	Beneficial	0.0029
EU	P11	*RdRp*	4192 (1398)	GAG-AAG	E-K	Neutral	0.0607
EU	P11	*TGB2*	130 (44)	CGT-GGT	R-G	Neutral	0.0292
EU	P11	*TGB3*	68 (23)	TTA-TCA	L-S	Neutral	0.1023
EU	P11	*CP*	463 (155)	GAA-AAA	E-K	Deleterious	0.0171
EU	P11	*CP*	497 (166)	GAT-GGT	D-G	Neutral	0.6633
EU	P11	*CP*	(155/166)	GAA-AAA GAT-GGT	E-K D-G	Beneficial	0.0030

^a^First number indicates the genomic nucleotide position; number in parenthesis indicates the amino acid in the corresponding protein

^b^Effects were classified as beneficial, neutral or deleterious according to the result of the 2-samples *t*-tests comparing the fitness values obtained for each mutant to the values measured for the non-mutated parental virus. *P*-values are reported in the last column

### *In vitro* RNA transcription

The resulting plasmids carrying the unique nucleotide substitutions were used to produce RNA transcripts using the mMESSAGE mMACHINE Kit (Ambion, Austin, USA), according to the manufacturer’s protocol. The RNA transcripts (at a final concentration of ca. 4 μg/μl) were used to inoculate *Solanum lycopersicum* L. cv. Beta Lux plants in an amount of 5 plants per variant. All plants used for experiments were grown in separated, insects-free, greenhouse cabins. For each plant, 10 μl of RNA was added (approximately 3.3 μl per each Carborundum-dusted leaf). PepMV-P22 is often asymptomatic on tomato plants whereas *Nicotiana benthamiana* Domin plants infected with PepMV displayed severe malformation, bubbling and discoloration, thus being a good indicator of infection and symptoms. In order to confirm the infectivity of constructed mutants *N. benthamiana* plants were also inoculated with all RNA transcripts. Each time five plants were used per variant. The symptoms on plants were monitored for 28 days post inoculation (dpi) and rated in a scale of 0–5 as follows: 0 – asymptomatic infection, 1 – mild mosaic, 2 – mosaic and malformation, 3 – severe malformation, 4 – yellow mosaic and chlorosis, 5 – necrosis. The entire experiment was conducted under greenhouse conditions (22–23 °C, 16 h photoperiod, 50% humidity) in a closed, monitored cabin. In parallel, five *S. lycopersicum* cv. Beta Lux and five *N. benthamiana* plants were inoculated with the phosphate buffer and used as controls for unwanted cross-contaminations. None of these control plants ever showed symptoms of infection.

### Virus quantification

From all of the tomato plants infected by different virus variants, total RNAs were extracted from the apical parts of the plants using the RNeasy Mini Kit (Qiagen, Hilde, Germany) according to the manufacturer’s protocol and dissolved in 30 μl of sterile water. RNAs were isolated 7, 14, 21, and 28 dpi, measured using a NanoDrop spectrophotometer (ThermoFisher Scientific, Waltham MA, USA) and diluted to the final concentration of ca. 100 ng/μl. First strand cDNAs were synthesized for each viral variant in five replicates using 1 μl of RNA at a final concentration of 100 ng/μl, Oligo(dT) primer (200 nM) and a RevertAid First Strand cDNA Synthesis Kit (ThermoFisher Scientific, Wilmington, DE, USA) according to the manufacturer’s protocol. To prepare a standard curve, cDNA from PepMV-P22 was synthesized using 1 μl of RNA at a final concentration of 1 μg/μl and the same protocol. cDNA obtained from PepMV-P22 was then serial 10-fold diluted from 1000 ng/μl to 1 pg/μl. The real-time quantitative PCR (qPCR) reaction was performed using FastStart Essential DNA Green Master containing SYBR Green I (Roche, Mannheim, Germany) and PepMVF1/PepMVR1 primers [52]. The relative amount of viral RNA in each sample at 7, 14, 21 and 28 dpi was calculated by comparing the obtained results to values from the standard curve from the two independent qPCR experiments, using LightCycler® 96 SW 1.1 software (Roche, Mannheim, Germany). In both qPCR experiments three technical replicates were performed per sample.

### Computation of fitness and statistical tests

Exponential growth rates were estimated from the PepMV genomic RNA accumulation data as follows. Let *n*_*t*_ be the number of PepMV genomes quantified by qPCR at sampling time *t* dpi. Then, we can define ${N}_t=\sum_{i=7}^t{n}_i$ as the cumulative number of genomes at *t* dpi. Assuming that the population is growing exponentially during the course of the experiment, then ${N}_t={N}_0{\mathrm{e}}^{rt}$, where *r* is the growth rate or Malthusian parameter. One can then infer *r* for each experiment by means of a logarithmic regression of *N*_*t*_ on *t*. These *r* values can be transformed into relative fitness (*W*) using the expression ${W}_i={r}_i/\left\langle {r}_0\right\rangle$, where *r*_*i*_ is the growth rate of mutant *i* (actually, of a given experimental replicate made for mutant *i*) and 〈*r*_0_〉 is the average growth rate estimated for the non-mutated strain.

The statistical significance of fitness effects associated to individual mutations compared to the fitness of the corresponding non-mutated strain was evaluated using 2-samples *t*-tests (reported in the last column of Table 1). Correction for multiple tests of the same null hypothesis was done using the Holm-Bonferroni sequential method. Otherwise indicated, confidence intervals will correspond to ±1 standard error of the mean (SEM).

Several statistical tests were performed using non-parametric Kruskal-Wallis or generalized linear models (GLM), the specific models are described when needed in the Results section below. All statistical tests were performed with IBM SPSS software version 23.

### Tests of epistasis

A subset of five mutations was introduced in the same positions of the same genes for both analyzed strains (RdRp/E1398K, TGB2/R44G, TGB3/L23S, CP/E155K, and CP/D166G). This test was performed to evaluate whether the fitness effect of these mutations depends on the genetic background where they appear, in other words, whether the effect of mutations was epistatic with the genetic background. A GLM with two orthogonal factors was fitted to the fitness data: *BACKGROUND* (CH2 and EU) and *SITE* (the five mutations tested in both backgrounds) and their corresponding interaction.

Mutations CP/E155K and CP/D166G were evaluated both alone and in combination (double mutant CP/E155K-D166G) in the isolate PepMV-P11 of the EU strain. Direct epistasis between mutations CP/E155K and CP/D166G was calculated as *ε*_155,166_ = *W*_*wt*_ *W*_155,166_ – *W*_155_ *W*_166,_ where *W*_*wt*_, *W*_155,156_, *W*_155_, *W*_166_ mean the fitness of non-mutated strain, double and single mutants.

## Results

### Observation of the symptoms on plants infected with different viral variants

A collection of single nucleotide nonsynonymous substitutions was introduced into the two viral genomes. In the case of four single nucleotide substitutions introduced into PepMV-P22 genome, symptoms were not observed and the virus was not detectable by RT-PCR. For all other viral variants, different disease symptoms were observed. The first symptoms on test plants were visible 7 dpi. Most of the variants with single mutation in viral genomes caused mild changes on leaf blades, such as deformation, slight discoloration, folding of the leaf blades and, at the later stage of infection, also growth retardation. In some of the cases, such as mutation CP/E2A (Table 1), the symptoms were stronger and severe deformation and discoloration of plants were observed. In the case of previously identified mutations: TGB3/K67E, CP/E155K and CP/D166G the symptoms were the same as expected: mutation TGB3/K67E caused necrotic changes on leaf blades, whereas the two mutations in the *CP* gene caused interveinal leaf yellowing symptoms on plants. Both, CH2 (PepMV-P22) and EU (PepMV-P11) strains showed the same symptoms as a result of the introduction of specific mutations.

### Dealing with the existence of lethal mutations

As mentioned in the previous paragraph, four mutants failed to generate a productive infection after three trials of the transcription-inoculation experiment (five replicates each). To check whether the reason is a lethal mutation or a failed inoculation experiments, additional experiments were performed. In the case of these four mutants, full genome sequences were obtained to verify the presence of additional mutations potentially responsible for lethal effects. Then, reverse-mutations to corresponding plasmid clones by site-directed mutagenesis were introduced, according to previously described procedures. The presence of the reverse mutation was verified by sequencing. The resulting plasmids carrying the reverse-mutation were used to perform transcription-inoculation experiments. In all of the cases, successful infection was observed, so we were confident that all mutations classified as lethal were not the result of failed inoculation experiments.

### Descriptive statistics of fitness effects associated to single nucleotide nonsynonymous substitutions

**Fig. 2:**
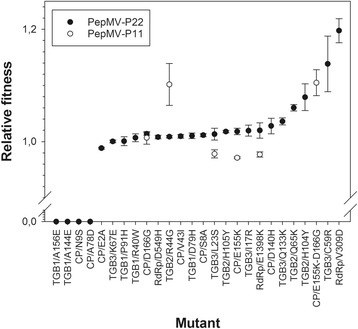
Fitness effects associated to each of the mutations listed in Table 1. Mutations have been ranked according to their effect. Error bars correspond to ±1 SEM

Figure 2 shows the distribution of fitness effects measured for the nonsynonymous mutations shown in Table 1. We have characterized the statistical properties of this DMFE on the PepMV-P22 mild isolate of the CH2 strain. The average fitness effect of the 25 mutations generated was 0.867 ± 0.078, with a median value of 0.898. Since the median is slightly larger than the mean, it may be that the distribution is asymmetric and skewed towards small fitness values; indeed, skewness tests shows a significant bias towards small fitness values (−1.911 ± 0.464; *t*_23_ = 4.119, *P* < 0.001) mostly due to the presence of four lethal mutations. The DMFE, has no excess of kurtosis (1.940 ± 0,902, *t*_23_ = 1.175, *P* = 0.252), indicating that most of the probability mass lies around the center and not in the extreme tail values. Since lethal mutations do not contribute to the next generation and are irrelevant from an evolutionary perspective, we have also computed the descriptive statistics of the DMFE excluding the four cases of lethality observed. The average fitness effect of the 21 viable mutations was 1.033 ± 0.011, with a median value of 1.014 that now is slightly smaller than the mean. Consistently, the DMFE of viable mutations is positively skewed (2.432 ± 0.501; *t*_19_ = 4.854; *P* < 0.001) due to the existence of beneficial mutations.

A non-parametric Kruskal-Wallis test shows that significant differences exist among mutations (*χ* ^2^ = 93.920, 23 d.f., *P* < 0.001). Next, we sought to evaluate which individual mutations significantly affected PepMV-P22 fitness. To do so, we compared the empirical fitness values obtained for each mutant to the values measured for the non-mutated virus. Two-samples *t*-tests, with sequential Holm-Bonferroni correction, showed that four mutations were lethal, one significantly deleterious (CP/E2A, *W* = 0.988 ± 0.002), 11 neutral and, surprisingly, nine beneficial (Table 1 shows the classification of each mutation into these categories and the associated *P* values). The beneficial fitness values ranged from 1.014 ± 0.002 for mutation CP/D166G (shown to be responsible for the induction of interveinal leaf yellowing symptoms) and 1.198 ± 0.021 for mutation RdRp/V309D. We must acknowledge that some cases classified as neutral may really be small effect deleterious or neutral cases and that we lack of enough statistical power to distinguish them from the null hypothesis of no fitness effects. However, a maximum likelihood estimation of the variance components shows that 99.40% of the total observed variance in fitness is attributable to true genetic differences among mutants and only a small 0.60% remains unexplained. Therefore, we are quite confident our numerical estimates are highly trustable.

### Differences among genes in the severity of mutational effects

Next, we sought to explore whether the effect of mutation on viral fitness was dependent upon which gene was mutated and in which base loci. To do so, we used GLM methods to fit the fitness data to a statistical model in which *SITE* was nested within *GENE*, and normally distributed errors and an identity link function were assumed (based on the lowest *BIC* value of this model). The model shows a highly significant effect of both *GENE* (likelihood-ratio test: *χ* ^2^ = 487.495, 4 d.f., *P* < 0.001) and *SITE* (likelihood-ratio test: *χ* ^2^ = 634.952, 20 d.f., *P* < 0.001), which supports the hypothesis that the effect of mutations depends on the gene where they appear and that differences among individual mutations affecting the same gene also exist.

Subsequently, *post hoc* sequential Bonferroni tests were run to more precisely seek for differences and similarities among genes. Mutations with the largest average beneficial effect (*W* = 1.075 ± 0.007) were those affecting the *RdRp* gene, followed by mutations affecting *TGB2* and *TGB3* genes, which were undistinguishable in terms of average effects (*W* = 1.041 ± 0.005). On average, mutations affecting *CP* gene were deleterious (*W* = 0.759 ± 0.004) but of significantly milder effect that mutations affecting the *TGB1* gene, which turned out to be the most deleterious ones (*W* = 0.604 ± 0.005).

### A first test of epistasis: mutational effects of given mutations depend on the strain wherein they appear

Next, we explored whether the fitness effect of a given nonsynonymous mutation may depend on the genetic background where it appears, thus indicating the existence of epistatic interactions, or conversely, fitness effects were independent on the genetic background. To this end, we focused in the set of five mutations that were generated in both strains (Fig. 2). Fitness data were fitted to a statistical model, by GLM, in which *SITE* and *BACKGROUND* were considered as orthogonal factors, with normally distributed errors and a link function (again, based in the lowest *BIC* among alternative models). Table 2 shows the results of this analysis. In short, the genetic background has no net effect on fitness (likelihood ratio test: *P* = 0.329) but it has an indirect effect via its interaction with *SITE* (likelihood ratio test: *P* < 0001), which indicates that the fitness effect of certain mutations is indeed dependent on the genetic background, but not the case for every mutation tested. The magnitude of this effect (measured using the ${\eta}_P^2$ statistic), is very large (>15%). This is clearly illustrated by Fig. 2, in which mutation TGB2/R44G has a much more beneficial effect on isolate PepMV-P11 than in PepMV-P22, whereas mutations CP/E155K, RdRp/E1398K and TGB3/L23S are slightly deleterious in PepMV-P11 while neutral in PepMV-P22.

**Table 2:** Results of the GLM analyses comparing fitness effects across two different PepMV strains (data in Fig. 2)

	*LRT* ^a^	d.f.	*P*	*η*_*P*_^2^ ^b^	1 − *β* ^c^	% variance^d^
Intercept	359.522	1	<0.001	0.999	1	0
*BAKGROUND*	0.953	1	0.329	0.019	0.137	0
*SITE*	25.972	4	<0.001	0.405	0.987	51.70
*BACKGROUND × SITE*	31.776	4	<0.001	0.470	0.998	48.30

^a^The likelihood ratio test statistic follows a *χ* ^2^ distribution

^b^Partial *η* ^2^ measuring the size of each effect when controlling for the other effects

^c^Power of the test

^d^Based on the maximum likelihood estimator of each component

Henceforth, these results support the existence of epistatic interactions between individual amino acid substitutions and the genetic background wherein they appear.

### Positive sign epistasis between mutations producing interveinal leaf yellowing symptoms

Two mutations have been described before as associated to the emergence of interveinal yellowing symptoms in PepMV-P22: CP/E155K and CP/D166G. Here we aim to explore the fitness consequences for a virus that may carry both mutations in its *CP* gene. To do so, we have evaluated the fitness effect of both mutations independently both in PepMV-P11 and PepMV-P22 and created the double mutant CP/E155K-D166G in PepMV-P11 and measured its fitness under identical experimental conditions. Mutation CP/E155K had a significant deleterious fitness effect on PepMV-P11 (0.971 ± 0.002; 2-samples *t*-test with Holm-Bonferroni correction: *P* = 0.017), whereas CP/D166G was effectively neutral in this strain (1.007 ± 0.011; 2-samples *t*-test with Holm-Bonferroni correction: *P* = 0.663). The fitness of the double mutant was *W* = 1.105 ± 0.023. Using the epistasis definition given in the Materials and Methods section, we calculated an epistasis coefficient between these pair of mutations of *ε*_155,166_ = 0.127 ± 0.036, which is statistically significant (*z* = 3.570, *P* < 0.001). A significant positive epistasis means that a PepMV-P11 virus containing both mutations together would be far more beneficial than the non-mutated virus and both single mutants. Furthermore, applying the mathematical conditions developed by Poelwijk et al. [53], we found that this epistatic interaction belongs to the so-called sign type, which means that the fitness effect of mutation CP/E155K was deleterious (negative) in a non-mutated PepMV-P11 genetic background but beneficial (positive) in a genetic background that already contains mutation CP/D166G. This creates certain degree of ruggedness in the fitness landscape around these mutations, with a low-fitness ridge involving mutation CP/E155K.

## Discussion

Knowledge about the DMFE is important for understanding the evolution of viral populations. It provides us information about the relationship between the sequences of the genome and viral fitness [54]. The fitness effect of mutations has been intensively studied for different plant and animal viruses [8–15]. It has been shown, that the effect varies between species or genomic regions and depends on environmental conditions [55, 56] (reviewed in [19]).

Here we explored the effect of single-nucleotide nonsynonymous substitutions on the fitness of a fast-evolving plant virus, PepMV. The fitness effect was evaluated using the virus’ natural host, *S. lycopersicum*. Random and non-random mutations were introduced into the genome of a mild isolate of the CH2 strain designated as PepMV-P22. Overall, 25 single-nucleotide substitution mutants were created by site-directed mutagenesis. In order to check the correlation between the mutational effect and the viral genetic background five mutations (CP/E155K, CP/D166G, RdRp/E1398K, TGB2/R44G, and TGB3/L23S) were also introduced into PepMV-P11 which is a mild isolate of the EU strain.

Non-random mutation in position TGB3/K67E, and both mutations in positions CP/E155K and CP/D166G, have previously been described as a determinant of disease symptoms [43, 45]. These mutations were first identified on tomato plants infected with the CH2 strain and still are present in the virus population. They have different effects on development of symptoms, virus replication and accumulation [43, 45, 57, 58]. Overexpression of both *TGB3* gene and the polymerase domain (POL) of the *RdRp* gene plays an important role in necrosis induction. The development of necrotic symptoms depends on virus accumulation level and can be modulated by tomato cultivar and environmental conditions [44]. Moreover, this single nucleotide substitution might have a great impact on the biological properties of the virus. At low temperatures, about 22–23 °C, necrotic isolates of the virus replicate faster and cause strong necrosis on plants, whereas at higher temperatures (above 25 °C) replication of these virus variants is slower and in consequence necrotic symptoms are not visible [44, 52]. Here we have shown that this necrotic mutation has a neutral effect on virus fitness. It might explain why a variant which cause severe changes on plants leading to death of the host, is still present in the population.

In the case of mutations CP/E155K and CP/D166G, a neutral or slightly beneficial effect has been found. The presence of these two mutations is connected with interveinal leaf yellowing symptoms, regardless of the temperature condition [43]. These mutations are the only factors involved in the formation of yellowing symptoms identified so far. It has been shown that yellowing symptoms tend to disappear after a few weeks in the upper parts of the plants [43]. Sequence analysis of multiple clones of *CP* gene of PepMV showed only the presence of wild-type sequence in the apical part of the plants. It suggests that selection pressure is acting on the advantage of wild-type virus [43].

The analysis of DMFE revealed that, in contrast to the previously studied viruses TEV and VSV [9, 12, 55], only a small fraction of lethal and deleterious mutations (20%) was found for PepMV. The size of the lethal fraction of mutations depends on various factors, such as genome organization of the virus (positive stranded, negative stranded, or ssDNA) or the host (bacteria, plants or animals) [19]. In other RNA viruses, the fraction of the lethal mutations ranges on average between 40 and 45% [9, 12]. Differences in mutational robustness between viruses may be the result of different genome architecture and contents [12]. In comparison with TEV [12, 13, 55], which is expressed as a polyprotein that subsequently cleavages into separate mature peptides, PepMV expresses five proteins, which are encodes in separate ORFs. For this reason, single mutations affecting one gene do not influence the other gene products, as it is the case for TEV.

PepMV molecular evolution rate was estimated around 5.57 × 10^−3^ nucleotide substitutions/site/year [59], although its actual rate of mutation has not been measured yet. It has been hypothesized that high mutation rates create a cloud of mutants at the population level, some of which may confer the viral quasispecies greater probability to evolve and adapt to ever-changing environmental conditions [60]. During PepMV genome replication, new genetic variants may arise with potentially beneficial effect on viral fitness, which bestow fast evolutionary population dynamics. Moreover, mutant spectra contribute to viral pathogenesis and are the target on which selection and genetic drift act to shape the long-term evolution of viruses [61]. Since PepMV was first detected on pepino in Peru [62], it has quickly spread all around the world. Moreover, the CH2 strain had almost completely replaced other strains in most parts of Europe and the United States. The more diverse a population, the more likely it may contain beneficial genetic variants with altered phenotypic properties, including virulence, pathogenicity or replicative capacity [63, 64]. Additionally, in recent years the emergence of many new, more aggressive variants has been witnessed in nature. Such a high amount of beneficial mutations which can be successfully maintained within the population causes serious problem in developing alternative plant protection methods and control strategies.

Overall, the analysis of constructed viral mutants revealed that the effect of mutations depends on the gene where they appear, although the differences between the mutations affecting the same gene are also significant. We found the strongest beneficial effect on viral fitness associated to amino acid replacements in the *RdRp*, whereas deleterious effects were associated to changes in the *TGB1* and *CP* genes. Viral *RdRp* gene contains three functional domains: methyl transferase, helicase and polymerase. None of the random mutations that we introduced into *RdRp* gene is located within the main functional domains and therefore do not affect its operation, yet may still affect the folding of the protein or its interactions with some unknown cell factor. In contrast, deleterious effect on *TGB1* could be related with its function. As it can mediate the suppression of signaling involved in systemic gene silencing, and thus in establishment of a systemic infection [65].

Moreover, we found that the effect of a mutation depends on the genetic background. It has been previously demonstrated that strains CH2 and EU, which show only 82% nucleotide sequence identity, clustered in separate groups on a phylogenetic tree [66]. It seems that their evolutionary history is different. Over the last few years, dramatic changes in the population structure of the virus have been observed. Strain EU, which was initially dominant in European crops, has been almost completely replaced by strain CH2, and currently remains only in mixed infections [38, 66]. A similar shift has been observed in the USA, where the population structure changed initially from US1 to EU, and subsequently from EU to CH2 [42]. It seems that the CH2 strain possesses a biological advantage over EU in temperate climate conditions. Moreover, strong expansion of CH2 strain promotes the arising of new viral variants. Perhaps these differences in mutational fitness effect result from divergent evolutionary paths and other adaptations.

In this study, we also performed a direct test of epistasis between a pair of mutations in positions CP/E155K (deleterious) and CP/D166G (neutral) introduced into the mild isolate PepMV-P11, observing an example of positive epistasis in the PepMV genome. In RNA virus, positive epistasis is quite more frequent than the negative epistasis [67]. Positive epistasis among deleterious mutations is a general feature of most small viruses, which are characterized by compact genomes, overlapping reading frames or multifunctional proteins [67]. The deleterious effects of different mutations can partially overlap and result in positive epistasis. Since mutation CP/E155K has a negative effect on fitness, it suggests that to produce viable virus each additional mutation has to necessarily exert little influence [67, 68]. Indeed, the deleterious effect of this mutation becomes significantly beneficial when it appears in presence of mutation CP/D166G, resulting in a case of sign epistasis [53]. Sign epistasis creates ruggedness in adaptive fitness landscapes [53], but does not precludes evolution to proceed from the wild type strain to the double mutant as the neutrality of mutation CP/E155K generates an accessible ridge that evolving population may traverse.

## Conclusions

The data obtained in our work revealed that PepMV possesses a surprisingly high tolerance to the fixation of nonsynonymous mutations. This mutational robustness may be associated, under certain circumstances, to a high evolvability of the virus [69, 70]. Moreover, the fitness value of a given mutation depends on the strain of PepMV being studied. Therefore, it seems that the mechanisms governing evolution are not universal for all strains. In this context, it is hard to predict the evolution of one viral strain based on studies conducted on a different one [30, 71].

## References

[1] Steinhauer DA, Domingo E, Holland JJ (1992). Lack of evidence for proofreading mechanisms associated with an RNA virus polymerase. Gene.

[2] Sanjuán R, Nebot MR, Chirico N, Mansky LM, Belshaw R (2010). Viral mutation rates. J Virol.

[3] Domingo E (2000). Viruses at the edge of adaptation. Virology.

[4] Chao L (1990). Fitness of RNA virus decreased by muller ratchet. Nature.

[5] Duarte E, Clarke D, Moya A, Domingo E, Holland J (1992). Rapid fitness losses in mammalian RNA virus clones due to Muller ratchet. Proc Natl Acad Sci U S A.

[6] De la Iglesia F, Elena SF (2007). Fitness declines in *Tobacco etch virus* upon serial bottleneck transfers. J Virol.

[7] Elena SF, Carrasco P, Daròs JA, Sanjuán R (2006). Mechanisms of genetic robustness in RNA viruses. EMBO Rep.

[8] Elena SF, Moya A (1999). Rate of deleterious mutation and the distribution of its effects on fitness in *Vesicular stomatitis virus*. J Evol Biol.

[9] Sanjuán R, Moya A, Elena SF (2004). The distribution of fitness effects caused by single-nucleotide substitutions in an RNA virus. Proc Natl Acad Sci U S A.

[10] Acevedo A, Brodsky L, Andino R (2014). Mutational and fitness landscapes of an RNA virus revealed through population sequencing. Nature.

[11] Visher E, Whitefield SE, McCrone JT, Fitzsimmons W, Lauring AS (2016). The mutational robustness of *Influenza A virus*. PLoS Pathog.

[12] Carrasco P, de la Iglesia F, Elena SF (2007). Distribution of fitness and virulence effects caused by single-nucleotide substitutions in *Tobacco etch virus*. J Virol.

[13] Bernet GP, Elena SF (2015). Distribution of mutational fitness effects and of epistasis in the 5′ untranslated region of a plant RNA virus. BMC Evol Biol.

[14] Domingo-Calap P, Cuevas JM, Sanjuán R (2009). The fitness effects of random mutations in single-stranded DNA and RNA bacteriophages. PLoS Genet.

[15] Peris JB, Davis P, Cuevas JM, Nebot MR, Sanjuán R (2010). Distribution of fitness effects caused by single-nucleotide substitutions in bacteriophage f1. Genetics.

[16] Keightley PD, Ohnishi O (1998). EMS-induced polygenic mutation rates for nine quantitative characters in *Drosophila melanogaster*. Genetics.

[17] Keightley PD, Davies EK, Peters AD, Shaw RG (2000). Properties of ethylmethane sulfonate-induced mutations affecting life-history traits in *Caenorhabditis elegans* and inferences about bivariate distributions of mutation effects. Genetics.

[18] Koufopanou V, Lomas S, Tsai IJ, Burt A (2015). Estimating the fitness effects of new mutations in the wild yeast *Saccharomyces paradoxus*. Genome Biol Evol.

[19] Sanjuán R (2010). Mutational fitness effects in RNA and single-stranded DNA viruses: common patterns revealed by site-directed mutagenesis. Philos Trans R Soc B.

[20] Keightley PD, Lynch M (2003). Toward a realistic model of mutations affecting fitness. Evolution.

[21] Orr HA (2003). The distribution of fitness effects among beneficial mutations. Genetics.

[22] Miralles R, Gerrish PJ, Moya A, Elena SF (1999). Clonal interference and the evolution of RNA viruses. Science.

[23] Escarmís C, Dávila M, Charpentier N, Bracho A, Moya A, Domingo E (1996). Genetic lesions associated with Muller’s ratchet in an RNA virus. J Mol Biol.

[24] Phillips PC (2008). Epistasis - the essential role of gene interactions in the structure and evolution of genetic systems. Nat Rev Genet.

[25] De Visser JAGM, Krug J (2014). Empirical fitness landscapes and the predictability of evolution. Nat Rev Genet.

[26] Lalić J, Elena SF. Magnitude and sign epistasis among deleterious mutations in a positive-sense plant RNA virus. Heredity. 2012;109:71–7. 10.1038/hdy.2012.15 PMC3400743 22491062

[27] Lalić J, Elena SF (2015). The impact of higher-order epistasis in the within-host fitness of a positive-sense plant RNA virus. J Evol Biol.

[28] Hillung J, Cuevas JM, Elena SF (2015). Evaluating the within-host fitness effects of mutations fixed during virus adaptation to different ecotypes of a new host. Philos Trans R Soc B.

[29] Cervera H, Lalić J, Elena SF (2016). Effect of host species on the topography of the fitness landscape for a plant RNA virus. J Virol.

[30] Cervera H, Lalić J, Elena SF (2016). Efficient escape from local optima in a highly rugged fitness landscape by evolving RNA virus populations. Proc R Soc B.

[31] Blystad DR, van der Vlugt R, Alfaro-Fernandez A, Cordoba MD, Bese G, Hristova D, Pospieszny H, Mehle N, Ravnikar M, Tomassoli L, Varveri C, Nielsen SL (2015). Host range and symptomatology of *Pepino mosaic virus* strains occurring in Europe. Eur J Plant Pathol.

[32] Mumford RA, Metcalfe EJ (2001). The partial sequencing of the genomic RNA of a UK isolate of *Pepino mosaic virus* and the comparison of the coat protein sequence with other isolates from Europe and Peru. Arch Virol.

[33] Roggero P, Masenga V, Lenzi R, Coghe F, Ena S, Winter S (2001). First report of *Pepino mosaic virus* in tomato in Italy. Plant Dis.

[34] Cotillon AC, Girard M, Ducouret S (2002). Complete nucleotide sequence of the genomic RNA of a French isolate of *Pepino mosaic virus* (PepMV). Arch Virol.

[35] Maroon-Lango CJ, Guaragna MA, Jordan RL, Hammond J, Bandla M, Marquardt SK (2005). Two unique US isolates of *Pepino mosaic virus* from a limited source of pooled tomato tissue are distinct from a third (European-like) US isolate. Arch Virol.

[36] Pagán I, Córdoba-Selles MD, Martínez-Priego L, Fraile A, Malpica JM, Jorda C, García-Arenal F (2006). Genetic structure of the population of *Pepino mosaic virus* infecting tomato crops in Spain. Phytopathology.

[37] Ling KS (2007). Molecular characterization of two P*epino mosaic virus* variants from imported tomato seed reveals high levels of sequence identity between Chilean and US isolates. Virus Genes.

[38] Hanssen IM, Paeleman A, Wittemans L, Goen K, Lievens B, Bragard C, Vanachter A, Thomma B (2008). Genetic characterization of *Pepino mosaic virus* isolates from Belgian greenhouse tomatoes reveals genetic recombination. Eur J Plant Pathol.

[39] Hasiów B, Borodynko N, Pospieszny H (2008). Complete genomic RNA sequence of the Polish *Pepino mosaic virus* isolate belonging to the US2 strain. Virus Genes.

[40] Hanssen IM, Paeleman A, Vandewoestijne E, Van Bergen L, Bragard C, Lievens B, Vanacher ACRC, Thomma BPHJ (2009). *Pepino mosaic virus* isolates and differential symptomatology in tomato. Plant Pathol.

[41] Moreno-Pérez MG, Pagán I, Aragón-Caballero L, Cáceres F, Fraile A, García-Arenal F (2014). Ecological and genetic determinants of *Pepino mosaic virus* emergence. J Virol.

[42] Ling K, Li R, Bledsoe M (2013). *Pepino mosaic virus* genotype shift in North America and development of a loop-mediated isothermal amplification for rapid genotype identification. Virol J.

[43] Hasiów-Jaroszewska B, Paeleman A, Ortega-Parra N, Borodynko N, Minicka J, Czerwoniec A, Thomma BP, Hanssen IM (2013). Ratio of mutated versus wild-type coat protein sequences in *Pepino mosaic virus* determines the nature and severity of yellowing symptoms on tomato plants. Mol Plant Pathol.

[44] Sempere RN, Gómez-Aix C, Ruiz-Ramon F, Gómez P, Hasiów-Jaroszewska B, Sánchez-Pina MA, Aranda MA (2016). *Pepino mosaic virus* RNA-dependent RNA polymerase POL domain is a hypersensitive response-like elicitor shared by necrotic and mild isolates. Phytopathology.

[45] Hasiów-Jaroszewska B, Borodynko N, Jackowiak P, Figlerowicz M, Pospieszny H (2011). Single mutation converts mild pathotype of the *Pepino mosaic virus* into necrotic one. Virus Res.

[46] Minicka J, Rymelska N, Elena SF, Czerwoniec A, Hasiów-Jaroszewska B (2015). Molecular evolution of *Pepino mosaic virus* during long-term passaging in different hosts and its impact on virus virulence. Ann Appl Biol.

[47] Hasiów-Jaroszewska B, Jackowiak P, Borodynko N, Figlerowicz M, Pospieszny H (2010). Quasispecies nature of *Pepino mosaic virus* and its evolutionary dynamics. Virus Genes.

[48] Eigen M, McCaskill J, Schuster P (1988). Molecular quasi-species. J Phys Chem.

[49] Schneider WL, Roossinck MJ (2000). Evolutionarily related Sindbis-like plant viruses maintain different levels of population diversity in a common host. J Virol.

[50] Legg JP, Thresh JM (2000). *Cassava mosaic virus* disease in East Africa: a dynamic disease in a changing environment. Virus Res.

[51] Hasiów-Jaroszewska B, Borodynko N, Pospieszny H (2009). Infectious RNA transcripts derived from cloned cDNA of a *Pepino mosaic virus* isolate. Arch Virol.

[52] Hasiów-Jaroszewska B, Komorowska B (2013). A new method for detection and discrimination of *Pepino mosaic virus* isolates using high resolution melting analysis of the triple gene block 3. J Virol Methods.

[53] Poelwijk FJ, Tanase-Nicola S, Kiviet DJ, Tans SJ (2011). Reciprocal sign epistasis is a necessary condition for multi-peaked fitness landscapes. J Theor Biol.

[54] Dean AM, Thornton JW (2007). Mechanistic approaches to the study of evolution: the functional synthesis. Nat Rev Genet.

[55] Lalić J, Cuevas JM, Elena SF (2011). Effect of host species on the distribution of mutational fitness effects for an RNA virus. PLoS Genet.

[56] Vale PF, Choisy M, Froissart R, Sanjuán R, Gandon S (2012). The distribution of mutational fitness effects of phage ϕX174 on different hosts. Evolution.

[57] Hasiów-Jaroszewska B, Minicka J, Pospieszny H (2014). Cross-protection between different pathotypes of *Pepino mosaic virus* representing chilean 2 genotype. Acta Sci Pol Hortoru.

[58] Minicka J, Otulak K, Garbaczewska G, Pospieszny H, Hasiów-Jaroszewska B (2015). Ultrastructural insights into tomato infections caused by three different pathotypes of *Pepino mosaic virus* and immunolocalization of viral coat proteins. Micron.

[59] Gómez P, Sempere RN, Aranda MA, Elena SF (2012). Phylodynamics of *Pepino mosaic virus* in Spain. Eur J Plant Pathol.

[60] Vignuzzi M, Stone JK, Arnold JJ, Cameron CE, Andino R (2006). Quasispecies diversity determines pathogenesis through cooperative interactions in a viral population. Nature.

[61] Domingo E, Martíb V, Perales C, Grande-Pérez A, García-Arriaza Arias J (2006). Viruses as quasispecies: biological implications. Curr Top Microbiol Immunol.

[62] Jones RAC, Koenig R, Lesemann DE (1980). *Pepino mosaic virus*, a new potexvirus from pepino (*Solanum muricatum*). Ann Appl Biol.

[63] Domingo E, Sheldon J, Perales C (2012). Viral quasispecies evolution. Microbiol Mol Biol Rev.

[64] Lough TJ, Emerson SJ, Lucas WJ, Forster RLS (2001). Trans-complementation of long-distance movement of *White clover mosaic virus* triple gene block (TGB) mutants: Phloem-associated movement of TGBp1. Virology.

[65] Pospieszny H, Hasiów B, Borodynko N (2008). Characterization of two distinct Polish isolates of *Pepino mosaic virus*. Eur J Plant Pathol.

[66] Gómez P, Sempere RN, Elena SF, Aranda MA (2009). Mixed infections of *Pepino mosaic virus* strains modulate the evolutionary dynamics of this emergent virus. J Virol.

[67] Elena SF, Solé RV, Sardanyés J (2010). Simple genomes, complex interactions: epistasis in RNA virus. Chaos.

[68] Sanjuán R, Moya A, Elena SF. The contribution of epistasis to the architecture of fitness in an RNA virus. Proc Natl Acad Sci USA. 2004;101:15376–9. 10.1073/pnas.0404125101 PMC524436 15492220

[69] Elena SF (2012). RNA virus genetic robustness: possible causes and some consequences. Curr Opin Virol.

[70] Stern A, Bianco S, Yeh MT, Wright CF, Butcher K, Tang C, Nielsen R, Andino R (2014). Costs and benefits of mutational robustness in RNA viruses. Cell Rep.

[71] Elena SF, Lalić J (2013). Plant RNA virus fitness predictability: contribution of genetic and environmental factors. Plant Pathol.

